# Supplementary material for: Promoter replacement by genome editing creates gain‐of‐function traits in Arabidopsis
Source: Plant Biotechnol J. 2025 May 5;23(7):2908–10. doi: 10.1111/pbi.70123 (PMC12205873; doi:10.1111/pbi.70123)

## Slide 1
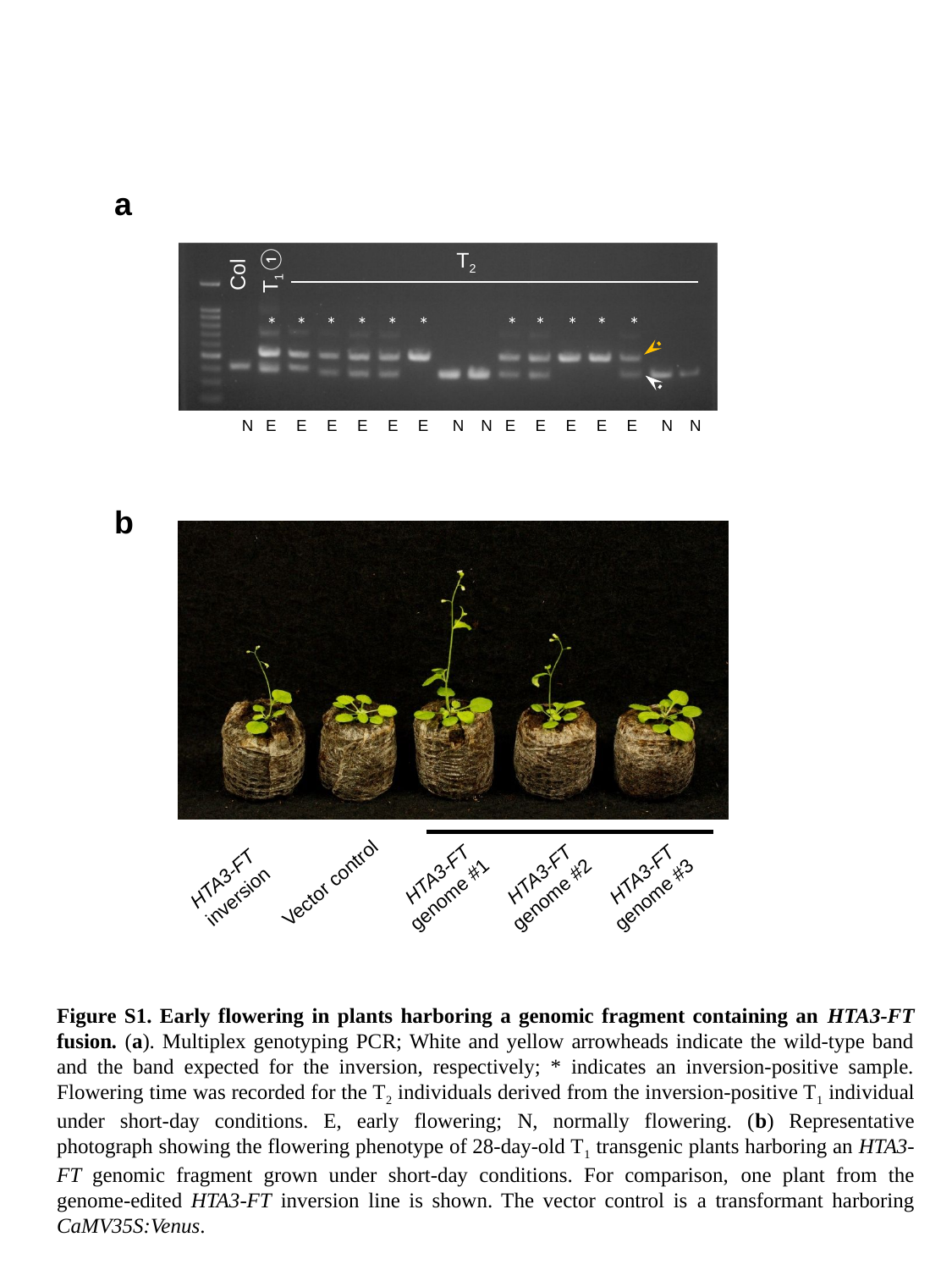

a
T2
T1①
Col
*
*
*
*
*
*
*
*
*
*
*
N
E
E
E
E
E
E
N
N
E
E
E
E
E
N
N
b
HTA3-FT
genome #1
HTA3-FT
genome #2
HTA3-FT
genome #3
HTA3-FT
inversion
Vector control
Figure S1. Early flowering in plants harboring a genomic fragment containing an HTA3-FT fusion. (a). Multiplex genotyping PCR; White and yellow arrowheads indicate the wild-type band and the band expected for the inversion, respectively; * indicates an inversion-positive sample. Flowering time was recorded for the T2 individuals derived from the inversion-positive T1 individual under short-day conditions. E, early flowering; N, normally flowering. (b) Representative photograph showing the flowering phenotype of 28-day-old T1 transgenic plants harboring an HTA3-FT genomic fragment grown under short-day conditions. For comparison, one plant from the genome-edited HTA3-FT inversion line is shown. The vector control is a transformant harboring CaMV35S:Venus.

## Slide 2
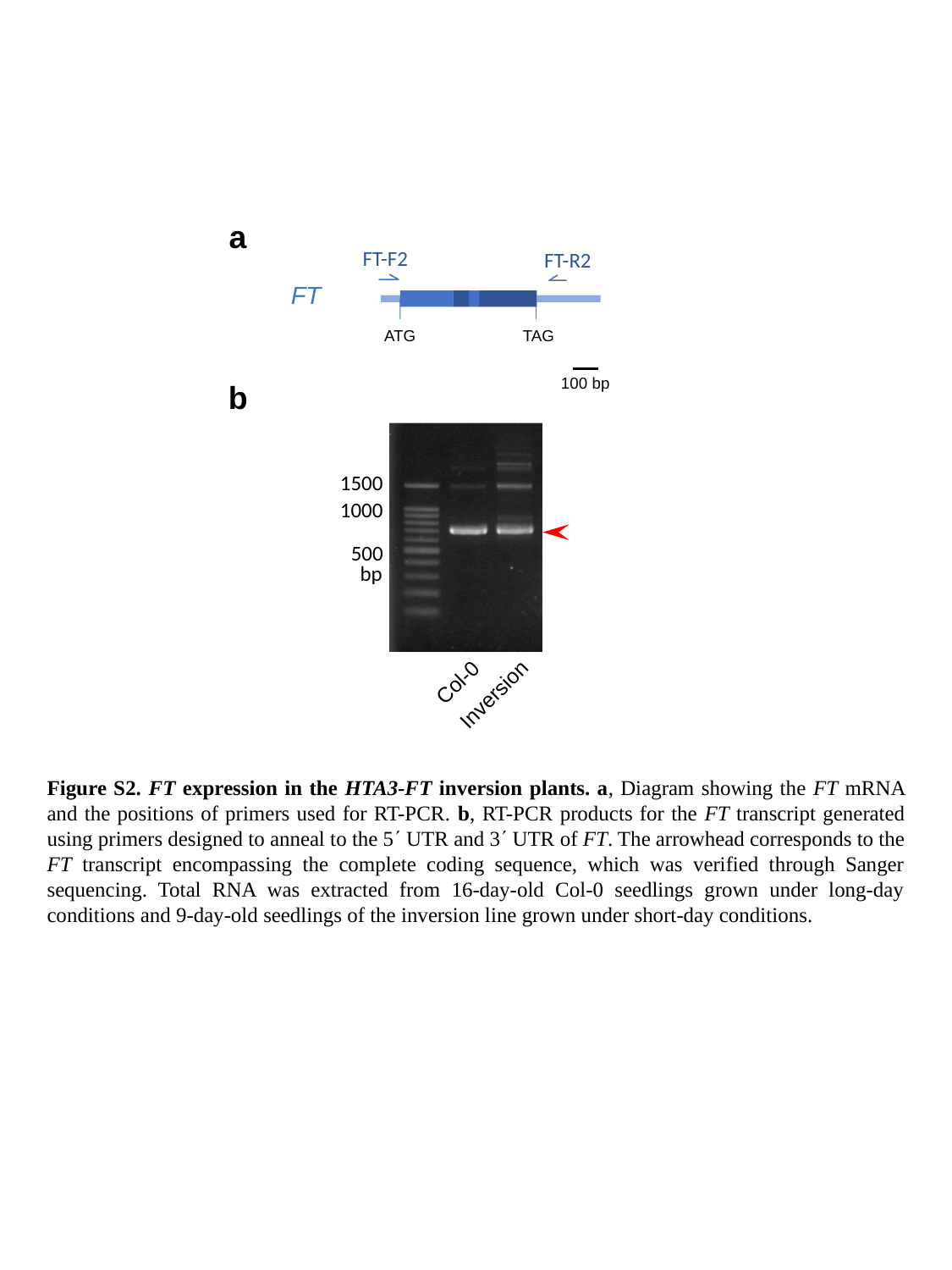

a
FT-F2
FT-R2
FT
ATG
TAG
100 bp
b
1500
1000
500
bp
Col-0
Inversion
Figure S2. FT expression in the HTA3-FT inversion plants. a, Diagram showing the FT mRNA and the positions of primers used for RT-PCR. b, RT-PCR products for the FT transcript generated using primers designed to anneal to the 5′ UTR and 3′ UTR of FT. The arrowhead corresponds to the FT transcript encompassing the complete coding sequence, which was verified through Sanger sequencing. Total RNA was extracted from 16-day-old Col-0 seedlings grown under long-day conditions and 9-day-old seedlings of the inversion line grown under short-day conditions.

## Slide 3
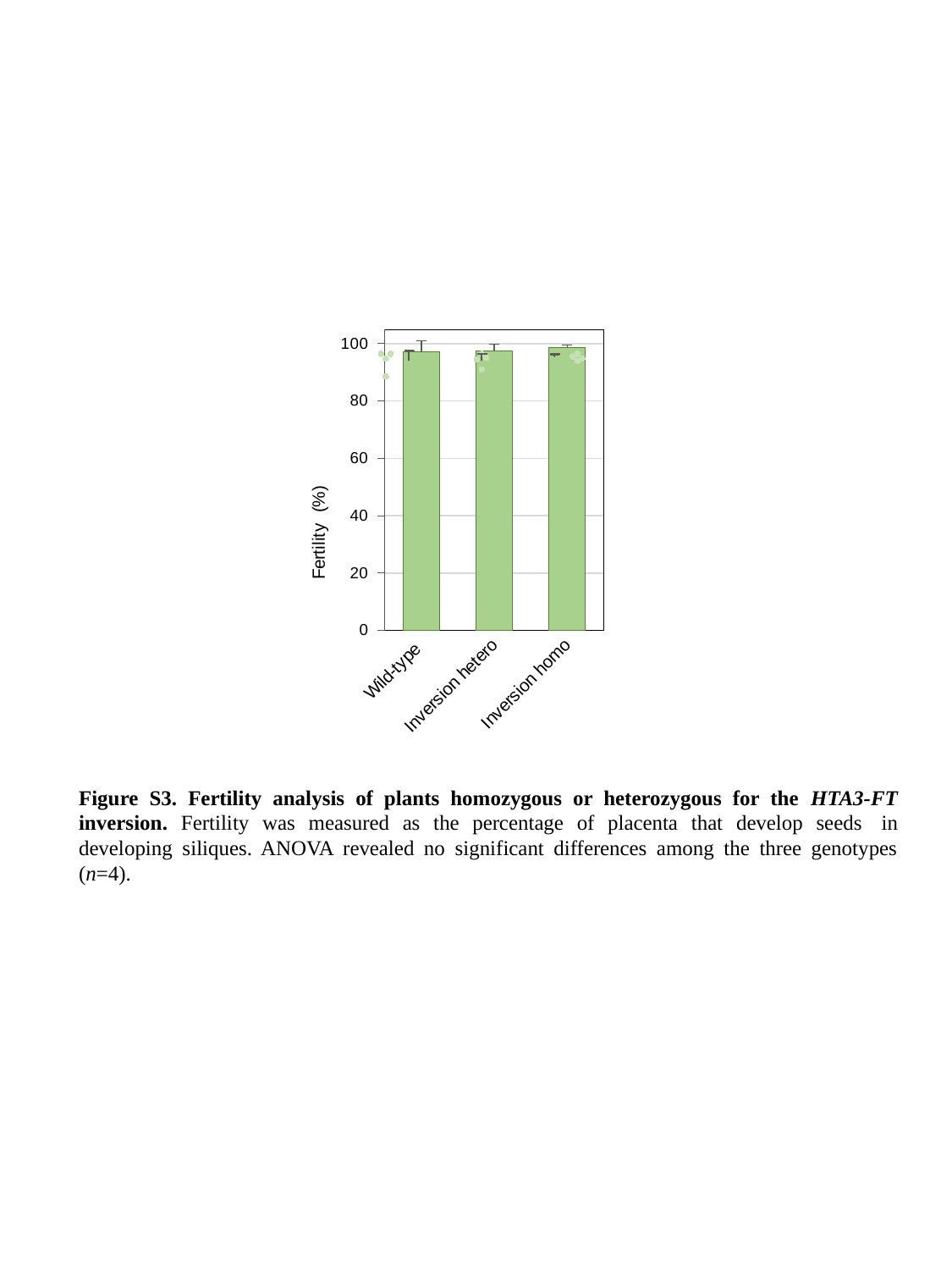

### Chart
| Category | |
|---|---|
### Chart
| Category | |
|---|---|
| Wild-type | 97.2475 |
| Inversion hetero | 97.47749999999999 |
| Inversion homo | 98.56 |Figure S3. Fertility analysis of plants homozygous or heterozygous for the HTA3-FT inversion. Fertility was measured as the percentage of placenta that develop seeds  in developing siliques. ANOVA revealed no significant differences among the three genotypes (n=4).

## Slide 4
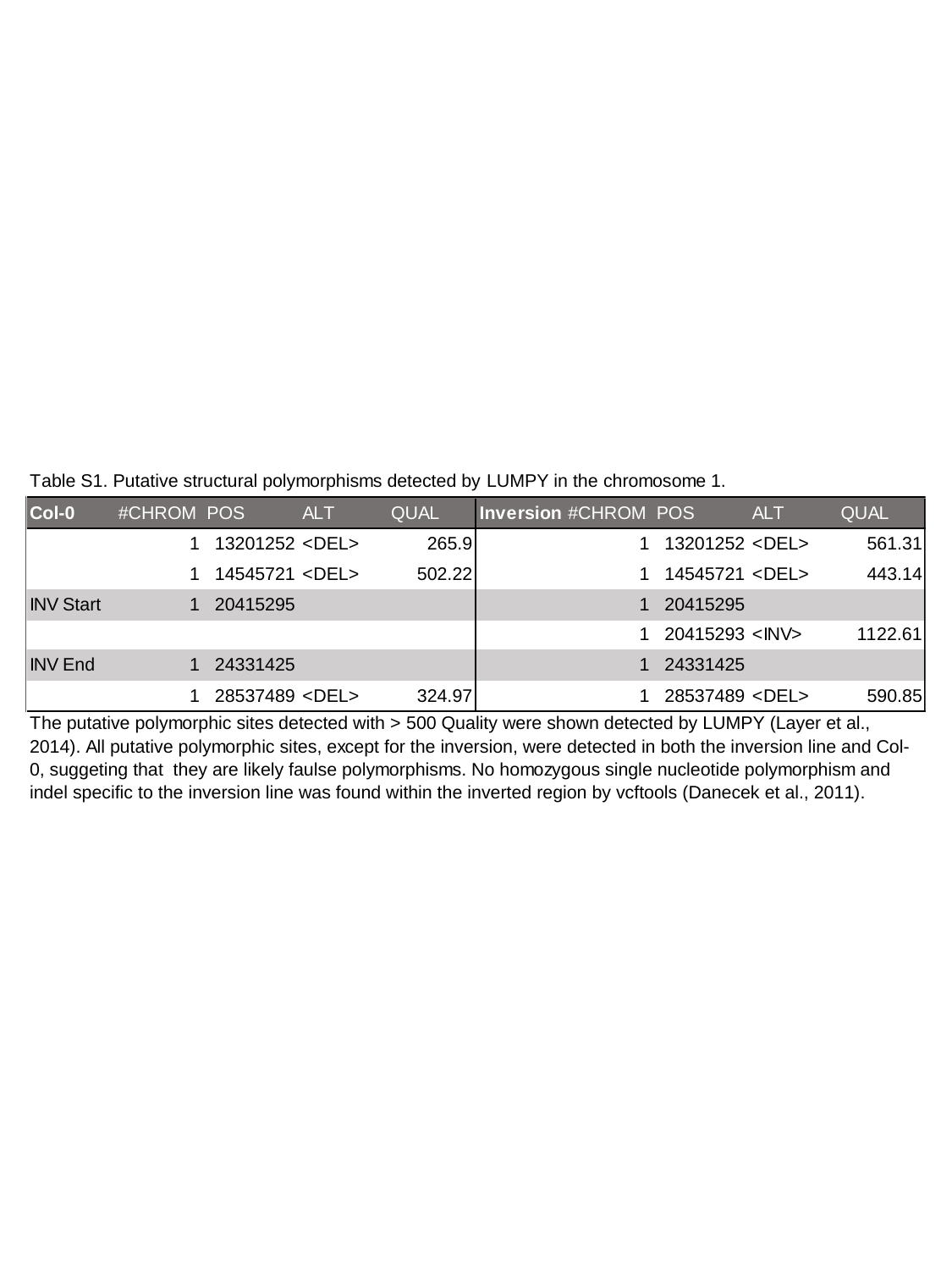

Supplement: Supplementary file 1 — Figure S1 Early flowering in plants harbouring a genomic fragment containing an HTA3‐FT fusion. Figure S2 FT expression in the HTA3‐FT inversion plants. Figure S3 Fertility analysis of plants homozygous or heterozygous for the HTA3‐FT inversion. Table S1 Putative structural polymorphisms detected by LUMPY in chromosome 1. [file PBI-23-2908-s001.pptx]
